# A Real-Time Magnetoencephalography Brain-Computer Interface Using Interactive 3D Visualization and the Hadoop Ecosystem

**DOI:** 10.3390/brainsci5040419

**Published:** 2015-09-30

**Authors:** Wilbert A. McClay, Nancy Yadav, Yusuf Ozbek, Andy Haas, Hagaii T. Attias, Srikantan S. Nagarajan

**Affiliations:** 1 Northeastern University and Lawrence Livermore National Laboratory, Boston, MA 02115, USA; E-Mails: yadav.na@husky.neu.edu (N.Y.); y.ozbek@neu.edu (Y.O.); 2 Dataura, Sierra Vista, Arizona, AZ 85635, USA; E-Mail: andy.haas@m-six.com; 3 Golden Metallic Inc., San Francisco, CA 94147, USA; E-Mail: htattias@goldenmetallic.com; 4 Biomagnetic Imaging Laboratory, Department of Radiology, University of California at San Francisco, San Francisco, CA 94122, USA; E-Mail: sri@radiology.ucsf.edu

**Keywords:** brain-computer interface, massive data management, machine learning algorithms, magnetoencephalographic (MEG), electroencephalography (EEG), 3D visualization, Hadoop Ecosystem

## Abstract

Ecumenically, the fastest growing segment of Big Data is human biology-related data and the annual data creation is on the order of zetabytes. The implications are global across industries, of which the treatment of brain related illnesses and trauma could see the most significant and immediate effects. The next generation of health care IT and sensory devices are acquiring and storing massive amounts of patient related data. An innovative Brain-Computer Interface (BCI) for interactive 3D visualization is presented utilizing the Hadoop Ecosystem for data analysis and storage. The BCI is an implementation of Bayesian factor analysis algorithms that can distinguish distinct thought actions using magneto encephalographic (MEG) brain signals. We have collected data on five subjects yielding 90% positive performance in MEG mid- and post-movement activity. We describe a driver that substitutes the actions of the BCI as mouse button presses for real-time use in visual simulations. This process has been added into a flight visualization demonstration. By thinking left or right, the user experiences the aircraft turning in the chosen direction. The driver components of the BCI can be compiled into any software and substitute a user’s intent for specific keyboard strikes or mouse button presses. The BCI’s data analytics of a subject’s MEG brainwaves and flight visualization performance are stored and analyzed using the Hadoop Ecosystem as a quick retrieval data warehouse.

## 1. Introduction

The use of brain-computer interfaces (BCIs), sometimes called mind-machine interfacing (MMI) or brain-machine interfacing (BMI), has been evolving for many years. These interfaces are used for both noninvasive procedures (such as magneto encephalography (MEG) and electroencephalography (EEG)) as well as for invasive procedures (such as electrocorticographic (ECoG) events). What follows is a brief discussion of the history and importance of BCIs in the noninvasive procedures of MEG and EEG as they relate to recent applications ranging from interactive video game technology to robotics and mobile applications.

One of the most dynamic current applications of these BCI developments is a video game called “Mind Balance” (created by researchers at the MIT Media Lab Europe and the University College of Dublin), which demonstrated how brain wave activity could be detected and measured without any need for wires, jacks, or plugs [1]. Instead of wires, the Mind Balance interface uses direct electroencephalography (EEG), cerebral data nodes, and Bluetooth wireless technology, all fitted into a sophisticated “Cerebus” headset to capture brain activity and feed it into a C# signal processing engine, which subsequently analyzes those signals and determines whether a subject is looking to the left or right. In this game, a frog-like character, “MAWG,” must be walked across a tightrope, using one’s mental focus.

Another dynamic BMI technology development was collaboration between Advanced Telecommunications Research Institute International (ATR) and Honda Research Institute (HRI) Japan. They developed a new “Brain-Machine Interface” (BMI) for manipulating robots, using brain activity signals. Their work has enabled the decoding of natural brain activity and the use of the derived data for the almost real-time operation of a robot, without an invasive incision of the head and brain. As a result, this technology is potentially applicable to other types of noninvasive brain measurements such as EEG and MEG. It is expected that such methods could yield the same result with less time lag and more compact BMI devices [2].

The current market trend centered on the integration of the gaming industry and Big Data analytics was approximated at $80 billion for 2014 [3,4,5,6,7,8,9,10,11,12,13]. The use of NoSql databases and the Hadoop Ecosystem yields keen competitive advantages over traditional relational transactional databases, moreover web-based games will become the go-to gaming platform and with the rapid adoption of mobile games [12]. Thus, an MEG based Brain-computer Interface (BCI) utilizing videogame analytics attracts two primary audiences: (1) the neuroscience and neuro-engineering scientific community, and (2) gaming and Big Data analytics industry. The market revenue for BCI applications interfaced to videogames has unparalleled future market revenue for avid gamers and diligent research scientists. In addition, the healthcare industry has now accepted gamification, or the utilization of game mechanics and design, to motivate people and influence their behaviors which is internally focused on wellness and healthy behaviors [12].

A current leader in BCI technology, lead by Emotiv Systems, is an innovative headset called Emotiv Epoc [14]. The Emotiv Epoc system measures the electrical activity associated with the brain and the muscles of the face, and it converts brain signals and activity into control signals. The Emotiv Epoch trains on acquired brain signals using the most common Artificial Neural Networks Learning and Training techniques, namely the Back Propagation algorithm and McCulloch-Pitts model [15].

**Figure 1:**
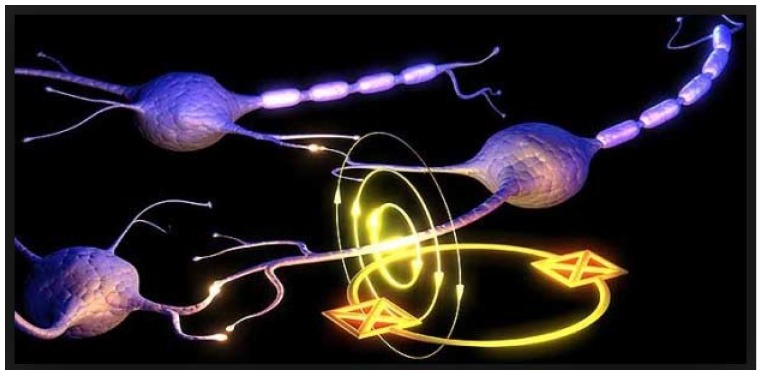
Superconducting Quantum Interference Devices (SQUIDS).

However, with respect to Big Data applications, MEG brain-wave data can exceed a terabyte in data storage per subject [16] as opposed to EEG brain-wave data typically with a maximum size of a few gigabytes. MEG provides signals with higher spatiotemporal resolution than EEG and typically results in improved signal properties (*i.e.*, lower signal to noise ratio) and increased BCI communication with less latency. MEG has higher spatiotemporal resolution due to better-designed and more expensive sensors called superconducting quantum interference devices (SQUIDS), illustrated in Figure 1.

Mellinger *et al.* demonstrated MEG has higher spatiotemporal resolution than EEG and results in better BCI communication speed [17]. Furthermore, Spuler, Rosenstiel, and Bogdan developed an MEG-Based Brain-computer Interface (BCI) using Adaptive Support Vector Machines, which outperformed non-adaptive machine learning classifiers on eight subjects with higher accuracies.

### 1.1. Scientific Literature Review of MEG/EEG and Hadoop

Previously, other research and computer scientists have utilized MapReduce and the Hadoop Ecosystem for parallel processing of massive EEG data sets. Moreover, Lizhe Wang *et al.* proposed the analysis of massive EEG data sets using the Ensemble Empirical Mode Decomposition (EEMD) neural signal-processing algorithm with MapReduce for data intensive computations to guarantee precision when neural signal data is used to classify and detect various brain disorders [18]. Another novel aspect utilizing the Hadoop Distributed File System is the Hadoop-BAM application presented by Niemenmaa *et al.*, where Hadoop-BAM is a unique and innovative library for the scalable manipulation of next-generation genomic sequencing data. The general consensus of most neuroimaging publications utilizing the Hadoop framework involves the integration of neuroimaging with genomic phenotypes, meaning linking the subject’s genetic information with the diagnosed neurological disorder displayed by the neuroimaging sensor (*i.e.*, Magnetic Resonance Imaging). Wang, Goh, and Montana utilized this approach with Alzheimer’s Disease Neuroimaging Initiative where they employed the usage of the Random Forest machine learning classifiers implemented into the MapReduce programming model and utilized the Hadoop Distributed File System for this mode of data acquisition [19].

### 1.2. Background

**Figure 2:**
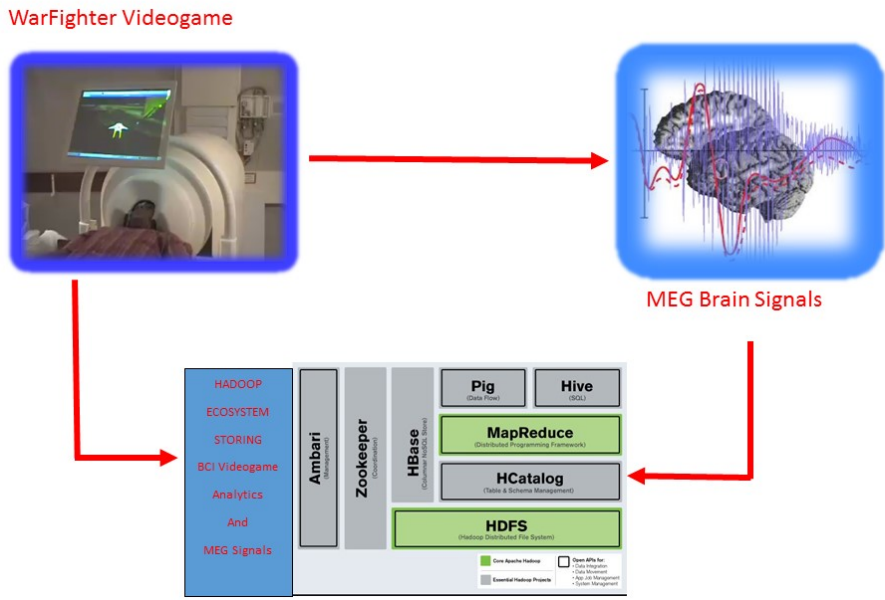
MEG BCI utilizing the Hadoop Ecosystem.

Mental operations occur in tens of milliseconds, and mental states (e.g., vigilance levels) that vary from seconds to minutes. In many respects, the optimal methods for monitoring these functions are EEG or MEG. Recent capabilities, developed for imaging cortical activity with MEG and EEG at a millisecond timescale, enable the identification of the most essential brain activity indices for different mental processes. Thus, we have developed a novel real-time BCI software application that classifies and translates a user’s brainwaves, converting their intent into a control action. Moreover, the storage and retrieval of MEG brainwave data and Hornet’s Nest flight simulator performance on large static data files is perfect for utilizing the Hadoop Ecosystem (Figure 2). Yu and Wang elaborate further and advocate the use of the Hadoop ecosystem as a means to distribute, store, manage and share the massive data and why it is an important issue [20].

The concept of converting brain activity into specific actions through the development of a novel BCI device suggests myriads of applications for military and civilian purposes, only some of which have been envisioned:

- Brain-machine interfaces
  ○ Pilots and flight control
  ○ Vigilance monitoring for air force, navy, or ground troop vehicles
  ○ Speech recognition [21]
  ○ Clinical settings: Monitoring patient mental states and providing feedback
  ○ Education: Improving vigilance, attention, learning, and memory
- Monitoring mental processes (“reading the mind”)
  ○ Detecting deception (FBI, CIA, other law enforcement agencies)
  ○ Predicting behavior
  ○ Detecting brain-based predispositions to certain mental tendencies (the brain version of Myers-Briggs)
  ○ Likelihood of improving with one type of training *versus* another
  ○ Likelihood of performing better under specific circumstances

### 1.3. Magneto Encephalography (MEG)

MEG is the primary process through which central nervous system (CNS) neuronal activity can be detected, catalogued, and analyzed. MEG identifies the very small magnetic fields that are created by infinitesimal electric currents flowing throughout CNS neurons during different mental activities. MEG essentially works because neuromagnetic signals penetrate the skull and scalp without being distorted. A magnetic source image (MSI) is created when MEG information is superimposed on a magnetic resonance image. The ability of the MEG process to identify mental activity with pinpoint accuracy is accomplished with the use of SQUIDS (superconducting quantum interference devices).

MEG provides a completely silent and noninvasive approach to gaining invaluable insight into the mechanics of the human brain. Further, MEG is now critically instrumental in helping doctors to positively affect patient lives by providing invaluable real-time neurological information useful in defining neurological disorders, planning surgical treatments, and detecting smaller zones of epileptic activity using greater precision (millimeter accuracy) than is currently available through standard EEG methods. Moreover, MEG-based monitoring systems (such as vital-sign monitoring products) have been developed to provide real-time information on brain activity that can be used in neuropharmacological investigations, trauma, and epileptic assessments, as well as pre-surgical functional mapping. In addition, the first fetal bio magnetic monitoring systems, which can monitor brain and heart activity *in utero*, have been developed and marketed for research purposes. Finally, MEG has become central to problem resolution in the diagnosis, evaluation, and treatment of

- Alzheimer’s disease
- Cognitive disorders (autism, learning disorders, Down syndrome)
- Mental disorders (schizophrenia, depression, dementia)
- Migraine headaches and chronic pain
- Multiple sclerosis
- Parkinson’s disease
- Stroke
- Traumatic brain injury
- Treatment of high-risk pregnancies

MEG became fast choice of imaging modality. Some of the most technologically advanced commercial MEG systems offer MEG sensor arrays of up to 275 distinct sensor channels with up to 128 simultaneous EEG sensors.

### 1.4. UCSF MEG System

**Figure 3:**
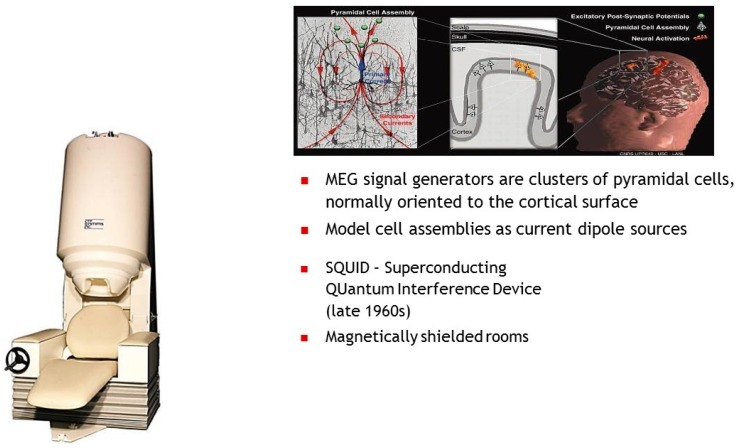
UCSF Magneto encephalography device using SQUIDS technology.

At the University of California, San Francisco (UCSF), MEG technology is being used to study multimodal and multiscale imaging of dynamic brain function as well as cortical spatiotemporal plasticity in humans [22]. For these studies, several novel algorithms had to be constructed, and UCSF had to fully utilize its twin 37-channel bio magnetometer. This machine uses SQUIDS-based detectors, housed in a magnetically shielded room (MSR), to noninvasively detect tiny magnetic fields generated by neuronal activity in the brain (Figure 3). From these signals, computational modeling allows a spatiotemporal view of the time course and spatial patterns of neuronal activity. The UCSF lab also uses digital 64-channel EEG and 3D computing facilities.

Finally, MEG technology is used in various departments at UCSF. The Magneto Encephalography Laboratory at UCSF is using the brain’s magnetic discharges, monitored by sensitive superconductive detectors, to isolate neocortical epilepsy prior to surgical resection [23]. These functional studies are combined with high-resolution magnetic resonance imaging (MRI) for diagnosis and surgical planning.

## 2. Experimental Section

Our goal was to identify and apply signal-processing methods coupled with machine-learning algorithms to rapidly extract the brain activity features of interest [24]. Ultimately, with these methods we were able to extract sufficient information within fractions of a second, thereby enabling us to monitor the ongoing flow of mental operations. This is an iterative process using Bayesian network (graphical models) machine-learning algorithms to extract information that is correlated with behavior and internal thought processes that are implied by the task conditions. The UCSF MEG scanner utilized a sampling rate of 1200 Hz using a low-pass hardware filter with a cut-off frequency at 300 Hz, thus the MEG scanner yielded an effective bandwidth of 300 Hz for the BCI experiment.

### 2.1. Brain-computer Interface Utilizing the VBFA Algorithm

We describe an implementation of Variational Bayesian Factor Analysis (VBFA) algorithms that have been applied to a successful identification of brain signal data. The implementation was used to replace mouse control of interactive visualization programs with control via computer interface. Specifically, we have inserted a simple left-*versus*-right control into a flight simulator environment, using MEG brain signal information in real time.

A training program (called VBFAgenerator) is invoked to condition the BCI to distinguish between the left and right responses of a given subject. VBFAgenerator concatenates the first 10 trials of MEG signal data [25] into rows of a data matrix. Each matrix row is the concatenation of samples for its respective MEG channel. For a MEG session, the total matrix size is 274 rows (channels) by 6010 columns (equal to the concatenation of 10 trials with 601 samples per trial). Each trial’s mean value is subtracted before data are added to the matrix. The VBFA generator then iterates the VBFA procedure until a maximum likelihood is reached. Matrix data sets representing the factors that describe the “essence” of MEG data are then saved.

VBFAgenerator is run separately for the left and right data sets, thus yielding a left and right set of factors. The factor sets are used in the real-time performance program (called VBFAperformer) to compute the likelihoods of each unknown trial with the left and right factors. The larger of the two likelihoods indicates the predicted response.

**Figure 4:**
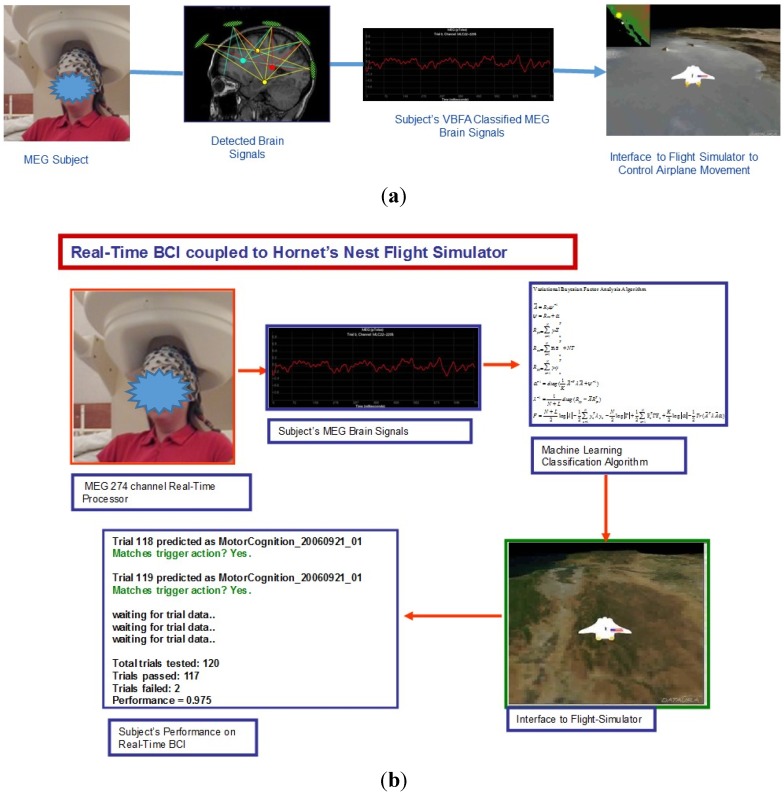
(**a**) MEG Brain Signals Classified with VBFAPerformer and Interfaced to Flight Simulator; (**b**) Real-Time BCI performance of predicted right and left thought-movement responses.

VBFAgenerator takes between 15 and 30 s to complete, and thereafter VBFAperformer runs side-by-side with the visualization application, analyzing the stream of trial data as it is made available from the MEG hardware to the PC. The process takes about 1/10 of a second to make a left-*versus*-right determination, which is then communicated as if it were a left or right button press in the flight simulator (Figure 4).

The current factor analysis algorithms yield about 95% accuracy in distinguishing left and right thought movement.

The accuracy of the real-time brain-machine interface is determined by comparing the actual thought responses of the subject to the predicted responses of the BCI. During a test session, the subject was equipped with left and right trigger buttons to be pressed in conjunction with the subject’s thoughts on moving left or right. These trigger bits are stored in a separate channel of the brain signal data. Real-time performance is computed by comparing these left or right trigger values with the predicted left or right responses (Figure 4b).

The BCI communicates with visualization by way of a pipe interprocess communication protocol [26]. The pipe protocol enables the BCI to operate independently of the visualization program. This process separation is particularly useful on dual-core processors, where the BCI runs parallel to the visualization, with no impact on the visualization CPU and graphics performance.

The pipe commands are single characters. For the left and right machine interface we have described here, the characters are a simple *L* and *R* [27]*.* A null character indicates that the BCI is finished sending commands. The receiving visualization program may then revert to the conventional mouse interface.

The machine-learning algorithm known as the Variational Bayesian Factor Analysis (VBFA) algorithm, shown in Equation (1) through Equation (10), was optimal for extracting different types of brain features because of the nature of the brain activity associated with particular types of mental processes. That is, the VBFA algorithm was tailored to the nature of the desired brain information acquired from a given subject.

Let *y*_n_ denote the signal recorded by sensor *I* = 1:*K* at time *n* = 1:*N.* The assumption corresponds to these signals arise from *L* evoked factors that are combined linearly. Let $x_{n}$ denote the signal of the evoked factor *j* = 1:*L*, at time *n*. Let *A* denote the evoked mixing matrix. The evoked mixing matrix contains the coefficients of the linear combination of the factors that produce the data. They are analogous to the factor-loading matrix in the factor analysis model [16]. Let $v_{n}$ denote the noise signal on sensor *i.* Mathematically, it follows from Equation (1) through Equation (10).

(1) $$y_{n}=Ax_{n}\;+\;v_{n}\;\left(\text{Bayesian Model}\right)$$

$v_{n}$ is sensor noise and $x_{n}$ are brain source signals

$p\left(x_{n}\right)=N\left(x_{n\;}|0,I\right)$ factors are zero-mean with unit precision

$p\left(v_{n}\right)=N\left(v_{n\;}|0,\lambda \right)$ noise is modeled by a zero-mean Gaussian with a diagonal precision matrix *λ*,

(2) $$p\left(y_{n\;}|x_{n}\right)=\;N\left(y_{n\;}|Ax,\lambda \right)$$

The distribution of the data conditioned on the factor is:

For the E-step in Factor Analysis, the posterior factorizers over time: $p\left(x_{n}|y_{n}\right)=\prod_{n=1}^{N}p\left(x_{n}|y_{n}\right)$

Inference Model: $\text{log }p\left(x_{n}|y_{n}\right)=\text{log }\left[\frac{p\left(y_{n\;}|x_{n}\right)p\left(x_{n}\right)}{p\left(y_{n}\right)}\right]$.

B. E-Step

The E-Step of VB-EM computes the sufficient statistics for the latent variables conditioned on the data.

For the post-stimulus period, *n = 1:N*, the latent variable are the evoked factors $x_{n}$. Compute their posterior mean $\bar{x_{n}}$ and precision Г by

(3) $$\begin{array}{c} \text{log }p\left(x_{n}|y_{n}\right)=\text{log }p{\left(x_{n}|y_{n}\right)}_{\;}+\;\text{log }p\left(x_{n}\right)-\text{log }p\left(y_{n}\right)\\ N\left(x_{n}|\mu ,Г\right)=\text{log }p{\left(x_{n}|y_{n}\right)}_{\;}+\;\text{log }p\left(x_{n}\right)-\text{log }p\left(y_{n}\right)\\ {\left|\frac{Г}{2\pi }\right|}^{\frac{1}{2\;}}e^{-\frac{1}{2\;}\left(x_{n}-\mu \right)}={\left|\frac{v}{2\pi }\right|}^{\frac{1}{2\;}}e^{-\frac{1}{2\;}{\left(y_{n}-Ax_{n}\right)}^{T}v\left(y_{n}-Ax_{n}\right)}+{\left|\frac{I}{2\pi }\right|}^{\frac{1}{2\;}}e^{-\frac{1}{2\;}{x_{n}}^{T}x_{n}}\\ {\left(x_{n}-\mu \right)}^{T}Г\left(x_{n}-\mu \right)={\left(y_{n}-Ax_{n}\right)}^{T}v\left(y_{n}-Ax_{n}\right)\;+\;{x_{n}}^{T}x_{n}\\ {x_{n}}^{T}Гx_{n}\;-{x_{n}}^{T}Г\mu \;-\;\mu^{T}Гx_{n}={y_{n}}^{T}vy_{n}\;-{y_{n}}^{T}vAx_{n}\;-\;{X_{n}}^{T}A^{T}vy_{n}\;+\;{X_{n}}^{T}A^{T}vAx_{n}\;+\;{x_{n}}^{T}x_{n}\\ \frac{\partial }{\partial \mu }=0\;\text{(To find the mean and precision, compute the gradient)} \end{array}$$

(4) $$\text{E-Step:}\;\mu \;=\;{Г}^{-1}A^{T}\lambda y_{n}$$

(5) $$Г=\;A^{T}\lambda A\;+I$$

C. *M-Step*

The *M-Step* of VB-EM computes the sufficient statistics for the model parameters conditioned on the data. We will divide the parameters into two sets. The first set includes the mixing matrix *A*, for which we compute full posterior distributions. The second set includes the noise precision ˍ and the hyperparameter matrix ˍ, shown by Equations (6)–(9).

M-Step

(6) $$\bar{l}\;=\;E\overset{N}{\underset{n=1}{\sum }}\left[\text{log }p\left(y_{n\;}|x_{n}\right)+\text{log }p\left(x_{n}\right)\right]=\frac{N}{2}\text{log }\left|\lambda \right|-\frac{1}{2\;}E\overset{N}{\underset{n=1}{\sum }}{\left(y_{n}-Ax_{n}\right)}^{T}\lambda \left(y_{n}-Ax_{n}\right)$$

(7) $$\begin{array}{c} \frac{\partial \bar{l}}{\partial A}=\;E\overset{N}{\underset{n=1}{\sum }}\left(y_{n}-Ax_{n}\right){x_{n}}^{T}=R_{yx}-AR_{xx}\\ R_{yx}=AR_{xx}\\ A=R_{yx}{R_{xx}}^{-1}\\ ∴\frac{\partial \bar{l}}{\partial A}=0,\lambda^{-1}\;=\frac{1}{N}diag\left(R_{yy}-AR_{xy}\right) \end{array}$$

Maximum of the posterior distribution by reconstructing the factors by the spatial graphical filter:

(8) $$\bar{x_{n}}={Г}^{-1}A^{T}\lambda y_{n}$$

The sufficient statistics of the factors, $R_{yx},$ and $\;R_{xx}$, and the data correlation matrix, $R_{xx}$, are given and Maximization of ˍ and ˍ yields

(9) $$\begin{array}{cc} \bar{A\;}=R_{yx}\psi^{-1}\;\psi =R_{xx}+\alpha & \text{Mixing Matrix of Observed Data \& Hidden Factors }\\ R_{yx}={\overset{N}{\underset{n=1}{\sum }}y_{n}{\bar{x}}^{T}}_{n}\;R_{xx}={\overset{N}{\underset{n=1}{\sum }}\bar{x_{n}}{\bar{x}}^{T}}_{n}+NT & \text{Sufficient Statistics for Data-Data Correlation \& Data Factor Correlations}\\ \alpha^{-1}\;=diag\left(\frac{1}{K}{\bar{A}}^{-T}\;\lambda \bar{A}\;+\psi^{-1}\right)\;\lambda^{-1}\;=\frac{1}{N+L}diag\left(R_{yy\;}-\bar{A}{R_{yx\;}}^{T}\;\right) & \text{Variance of “A” Mixing Matrix measured by Alpha.}\;\text{Variance of Noise matrix measured by lambda} \end{array}$$

The completed Variational Bayesian (VB) EM algorithm for the factor analysis model is shown in Equation (1) through Equation (10). Finally, the objective function that this algorithm maximizes is

(10) $$F\;=\frac{N+L}{2}\text{log }\left|\lambda \right|-\frac{1}{2}\overset{N}{\underset{n=1}{\sum }}{y_{n}}^{T}\lambda y_{n}-\frac{N}{2}\text{log }\left|Г\right|+\frac{1}{2}\overset{N}{\underset{n=1}{\sum }}{\bar{x}}^{T}Г\bar{x_{n}}+\frac{K}{2}\text{log }\left|\alpha \right|-\frac{1}{2}Tr\left({\bar{A}}^{T}\lambda \bar{A}\alpha \right)$$

### 2.2. Why Big Data Analysis for Healthcare and Brain-computer Interface Technology?

During operation of biomedical imaging sensors (e.g., MEG, functional Magnetic Resonance Imaging (fMRI)), large amounts of data are produced and processed for instance in decision making [18] regarding a surgical operation or a healthcare treatment plan. Since 2012, the global digital healthcare data was estimated to be equal to 500 petabytes and is presumed to reach 25,000 petabytes in 2020 [12].

The vast collection of biomedical imaging data encompasses a large collection of complex data sets, which are tedious to process using traditional relational database management systems and other standard data processing applications (*i.e.*, Matlab). Thus, we needed a framework-based system, which had puissant retrieval features to query specific data [18] and the data could be distributed across multiple computers in case of fault analysis. The Hadoop Ecosystem is the quintessential framework to hand the processing of massive biomedical datasets, and data replication across multiple computers to handle parallel processing of MapReduce algorithms and ameliorates system failures utilizing distributed computing. Hadoop is an open-source software platform, which designed to stored and process massive profusions of data, which would severely compromise one computer or server. Additionally, the Hadoop Ecosystem is composed of many different components such as Pig, HBase, Hive, Flume, Spark, and Mahout, which collaboratively can work together. Moreover, the ubiquitous concern regarding data security is a major dilemma to HIPAA regulations in the medical research field, thus utilizing the Hadoop Ecosystem major advancements have been designed to address data privacy issues utilizing open-source projects, such as Knox Gateway (contributed by HortonWorks) [28], which is a major advancement over the traditional Unix-based file system.

Although, for this paper the usage of five subjects for testing with each MEG CTF file being approximately a few gigabytes per left and right brain hemisphere per subject, does not constitute a typical Big Data problem in terms of size. The usage of the Hadoop Ecosystem provides an open-source coding environment capable of gaining sapience from complex, often time’s poor signal to noise ratio, and voluminous data. The Hadoop Ecosystem is capable of answering questions that were precedent as unanswered. Using the MapReduce paradigm and the myriad of applications in the Hadoop framework yields veracity to massive amounts of biomedical sensory data within milliseconds and fast querying and indexing of data utilizing its schema-less NoSql column-oriented database called HBase.

In the subsequent Section 2.3, we will illustrate and show why the Hadoop Ecosystem is the archetypal application for analyzing, storing, and answering various forms of impending healthcare data challenges. Moreover, the Hadoop Ecosystem is an excellent data store and analysis application for Brain-computer Interface technology, which includes many domains of research, for instance signal processing, machine learning, databases, and computer graphics.

### 2.3. Hadoop Ecosystem

Hadoop 1.0.0 was originally released by Apache in 2011, consisting of mainly the Hadoop Distributed File System (HDFS) and MapReduce. The Hadoop Platform soon became realized as an ecosystem, which is constantly evolving where each unit in the ecosystem facilitated a specific data analysis or data storage need. We selected the Hadoop Ecosystem as a data analysis warehouse because of its scalability, performance, and fault tolerance. The Hadoop Ecosystem represents data in terms of key/value pairs. The utilization of the Hadoop NoSql database, HBase, data is represented as a collection of wide rows. The atomic structures in HBase make global data processing using MapReduce and row-specific reading/writing using HBase undemanding [29].

**Figure 5:**
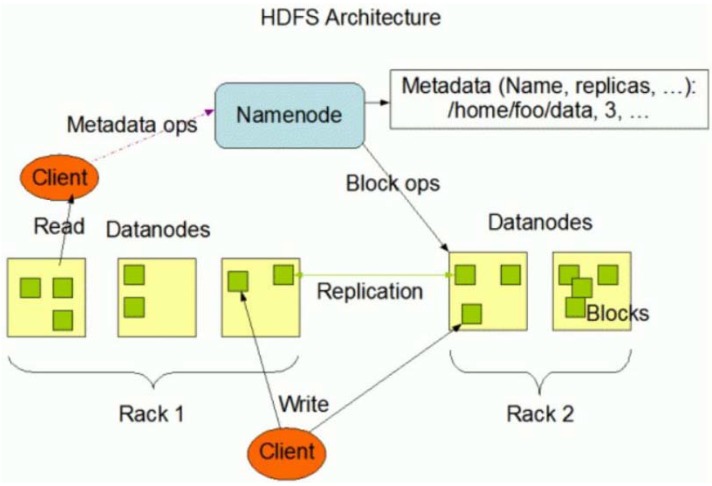
Hadoop Distributed File System Architecture.

**Figure 6:**
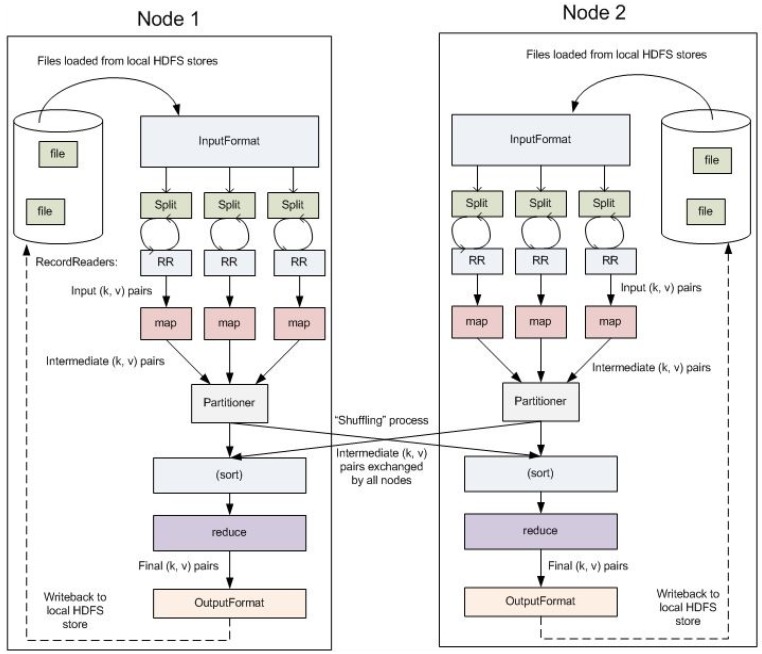
MapReduce Computational Paradigm Model.

For the purpose of the MEG BCI project we will focus on four different brief descriptions of the Hadoop Ecosystem:

1. Hadoop Distributed File System (HDFS)
2. MapReduce
3. HBase and Zookeeper
4. Pig
  1.) The Hadoop Distributed File System (HDFS) is a way to store and analyze large static data files across multiple machines as opposed to a single machine holding the entire disk capacity of the aggregated files. HDFS uses data replication and distribution of the data and is created to be fault-tolerant. A file is loaded into HDFS and is replicated and split into units called blocks, which are typically 64 MB of data and processed and stored across a cluster of nodes or machines called DataNodes. HDFS uses the Master and Slave architecture where the Master (NameNode) is responsible for management of metadata and execution of jobs to the DataNodes (Figure 5).
  2.) MapReduce is a computational paradigm for parallel processing using two sequences of execution. First the map phase is a set of key-value pairs and the necessary function is executed over the key-value pairs to produce another interposed key-value pairs. The last application is the reduce phase where the interposed key-value pairs are aggregated by a key and the values are combined together to a final reduction output (Figure 6). In Hadoop, files are split using an input format. An input split is a byte-oriented view of a chunk of the file to be loaded by a map task. Using MapReduce for medical and sensory imaging is becoming a tool of choice, particularly because medical imaging is multi-dimensional data which MapReduce can logically split the data into records and input splits correctly [30,31].

**Figure 7:**
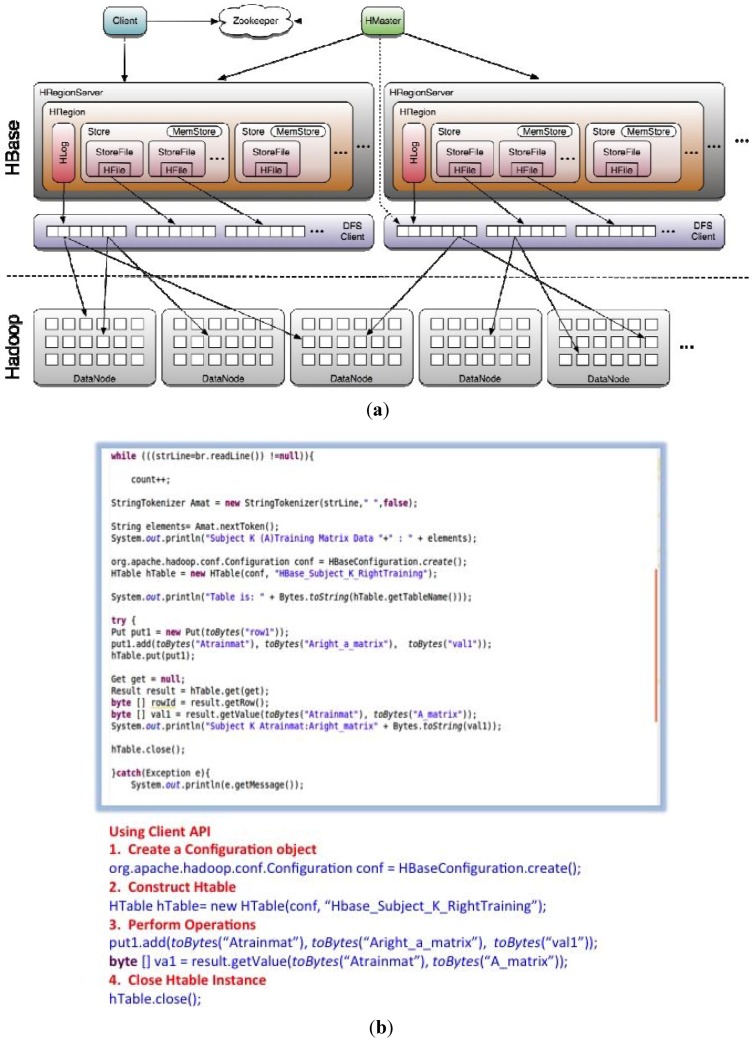

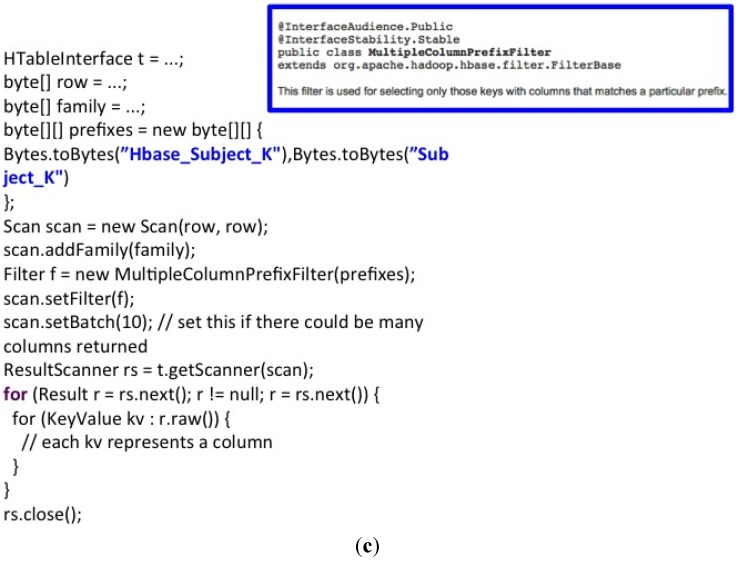
(**a**) HBase built on top of HDFS; (**b**) Java Client API of Subject K information into HBase; (**c**) Java Client MultipleColumnPrefixFilter of Subject K information into HBase.

  3.) HBase is a distributed column-oriented database built on top of HDFS to provide storage for Hadoop Distributed Computing using ZooKeeper as a service for maintaining configuration information of the HRegionServers shown in Figure 7a, based on a “master” and “slave” node architecture.

In the BCI project, HBase was preferred for data storage over HDFS for analysis of the VBFA training matrices files for each subject’s MEG brainwave data, which were examined for fast record lookups, updates, sparse column family data model (*i.e.*, large sparse singular value decomposition matrices), and the combined maximum data size of all subjects was less than a petabyte of storage. We illustrate an example data flow diagram of a subject’s training matrices being implemented into HBase and using Zookeeper for maintaining configuration information (Figure 7b). In Figure 7b, statement 1, create a configuration object that seeds information to establish a client connection; in statement 2, we create a table name: HTable (conf, “Hbase_Subject_K_RightTraining”); statement 3, we perform the necessary operations by using a put statement which saves a operation for a single row: Put1.add (*toByte*s (“Atrainmat”), *toBytes*(“Aright_a_matrix”), *toBytes*(“val1”)); statement 4, we close the HTable instance with: HTable.close().

In Figure 7c, we utilize Client Request Filters which use Get and Scan instances which can be optionally configured with filters and applied on the HBase RegionServer. Using MultipleColumnPrefixFilter class allows specifying multiple prefixes. For instance in Figure 7c, all columns in a row and family that start with “HBase_Subject_K” or “Subject_K”, the benefit of using the HBase Filter Classes allows for fast scanning and indexing on discontinuous sets of columns from very wide rows. This approach allows fast and quick scans for analyzing subject’s training data and relevant metadata from HBase ColumnFamilies describing for instance the subject’s trials, performance, and MEG scanner positioning [32].

**Figure 8:**
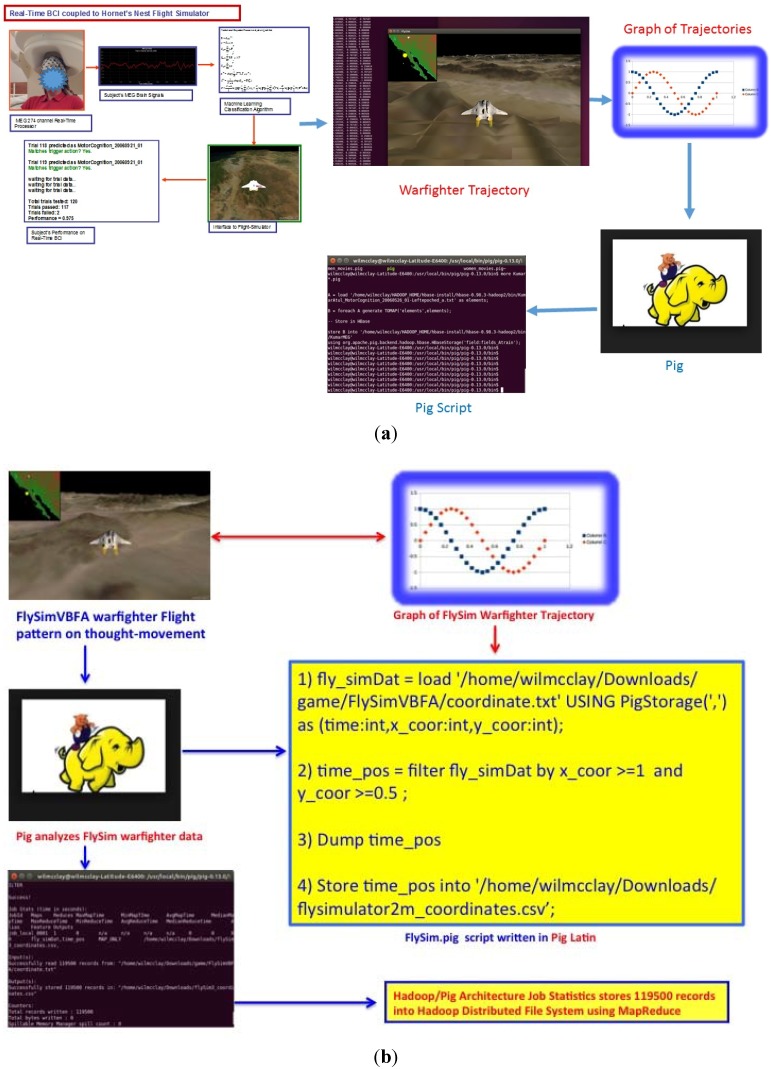
(**a**) Pig Scripts to analyze the warfighter’s trajectory based on subject’s thought movement; (**b**). A step-by-step analysis of the FlySimVBFA.pig script with MapReduce storing 119,500 warfighter trajectory records into the Hadoop Distributed File System.

  4.) Pig is a simple-to-understand, novel, and elegant data flow language used in the analysis of large data sets. Additionally, Pig is a higher-layer of abstraction of MapReduce and the Pig system deciphers the higher-level language into a sequence of MapReduce jobs [30]. The benefits of using Apache Pig is its ease and applicability to analyzing unstructured data, for instance MEG SQUID sensors which can fail during real-time processing while playing the BCI warfighter simulator. Moreover, in Figure 8, we used Pig for ETL (Extraction Transformation Load) processing of videogame analytics as an underpinning of Pig exemplary power as a data flow-language.

A Pig Script written in the language, Pig Latin, which is automatically converted into MapReduce jobs by the Pig interpreter. For the MEG BCI project we used Pig to process the warfighter videogame analytics as a basic example of Pig. Pig scripts were written for example to analyze the trajectory of the warfighter as the user’s thought movement guided the warfighter either left or right based on the VBFA’s classified brainwaves (Figure 8a). The main benefit of using Pig for this BCI project was for quick and arbitrary warfighter videogame processes for structured and unstructured user feedback data and significantly decreases development time. Additionally, Pig scripts are automatically converted into MapReduce jobs the Pig interpreter, so you can analyze the data in a Hadoop cluster even if you are not familiar with MapReduce. In Figure 8b, we illustrate the series of steps used for writing the Pig based MapReduce script shown in Figure 8a. In Figure 8b, the FlySimVBFA.pig is an Apache Pig script is written in Pig’s language, Pig Latin, which is a facile and lucid query algebra that allows the user to express data transformations such as filtering, merging data sets, and/or applying functions to records or groups of records. The perks of using Pig Latin is a simple to understand data flow language for analysts or novice programmers whom are familiar with scripting languages (*i.e.*, Matlab or IDL), second it is a agile and iterative language with a robust MapReduce compilation engine which allows for multivalued and encapsulated operations performed on immense data sets. The analysis of the FlySimVBFA.pig script in Figure 8b, is below:

1) fly_simDat = load “/home/wilmcclay/Downloads/game/FlySimVBFA/coordinate.txt” USING PigStorage(“,”) as (time:int,x_coor:int,y_coor:int);

Line 1 uses the *load* statement that returns a *tuple*. Each tuple has multiple elements, which can be referenced by position or by name and stores the coordinate.txt file using the *PigStorage* field-delimited text format or PigStorage(“,”).

2) time_pos = filter fly_simDat by x_coor >= 1 and y_coor >= 0.5;

Line 2 uses the *filter* operator to work with the tuples or rows of the data.

3) DUMP time_pos

Line 3 uses the *DUMP* alias to display the content of a relation or in our case, time_pos. However, a user should note that the relation should be confined to fit on the console screen, otherwise use the *LIMIT* operation on the alias for a more accurate display.

4) Store time_pos into “/home/wilmcclay/Downloads/flysimulator2m_coordinates.csv”;

Line 4 uses the *STORE* alias to store data from a relation or *“time_pos”* into a directory, and Pig will create the directory *“flysimulator2m_coordinates.csv”* and store the relation in the file named part-nnnn in the *“flysimulator2m_coordinates.csv”* directory.

## 3. Results

A major difficulty in current BCI systems is that MEG (and other modalities such as EEG and fMRI) data are highly variable because the brain does many different things at the same time, most of them unrelated to the task at hand. For example, when focusing on making the cursor move to the right, a subject also hears ambient sounds, sees a picture on the wall, and feels an aching muscle from the gym. Thus, it can be difficult to localize the subject’s intended command, because the resulting brain activity from unrelated tasks interferes with the signal we wish to localize.

During MEG scanning both for training and test trials, we routinely fit the subjects’ head in the scanner snugly with cushion pads, which permit minimal movement. Furthermore, we did all our experiments with subjects lying supine, which we found to minimize head-movements during scans. We also measure head position before and after each run in the scanner and reject any data set where the subject’s movement is greater than 5mm. These experimental procedures ensure that head movements are minimal during the data collection. Head movements for each block of trials was within 5 mm and subjects typically move not more than 1–2 mm over the course of a block. Eye movements were not monitored. Large eye movements could be picked up by the MEG sensors, but during “training” trials subjects were typically fixating on the screen and did not have eye movements.

Finally, since there is no time delay between training trials and test trials (*i.e.*, flight simulator), it is unlikely that systematic shifts in head movement could occur between these time periods. Therefore, we are convinced that head movements cannot account for our results.

Our techniques at the UCSF MEG Laboratory are based on probabilistic hidden variable models, which describe the observed MEG sensor data in terms of underlying unobserved brain sources. These models can handle the variance of the data caused not just from interference source activity but also from electrode position, across-subject variability (brains respond differently to the same stimulus), and within-subject variability [33]. Consequently, we can extract features of the MEG data that lead to more accurate classification.

**Figure 9:**
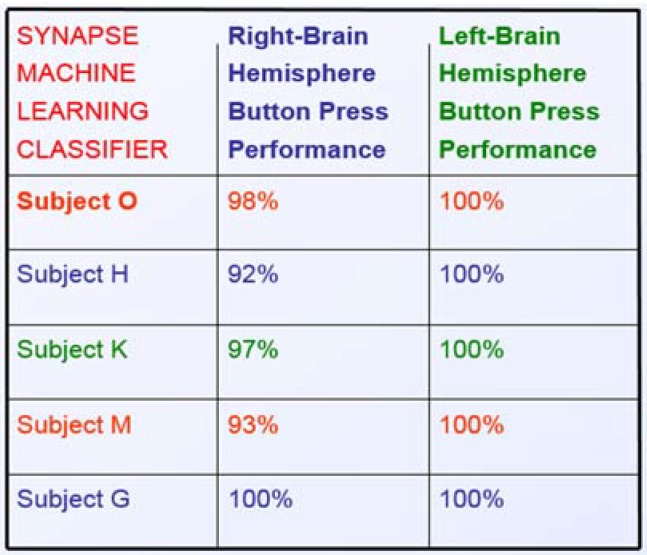
Five Subject’s Performance.

We tested the performance of the VBFA algorithm on five subjects scanned on a 274-channel MEG sensor array for classification of right and left button-press movement (Figure 4b). Each subject was given a sequence of ten trials for training the VBFA algorithm parameters on the right and left neuromotor movement of the brain. After the training trials, the VBFA algorithm was tested for performance in real time on a subject’s arbitrary trial of either a right or left neuromotor button-press movement. Performance was computed using the first 60 trials during each session. The known trigger value (representing the actual button press) was compared with the predicted left/right response, and a percentage of accuracy was recorded after the 60 trials. The five MEG subjects, who performed over 90% on the neurological movement testing, demonstrated the excellent performance and robustness of the VBFA algorithm. The subjects’ performance data are shown in Figure 9.

## 4. Conclusions

We are currently extending in several directions the machine-learning module that infers user intent from data. We delineate two of these directions here.

First, the classification algorithm described in this paper is based on modeling the data as i.i.d. Gaussian (conditioned on the mixing matrix). However, real MEG data are non-Gaussian and exhibit strong temporal correlations. A model that accounts for those features would describe the data more accurately and could therefore lead to improved classification and performance. We are exploring several extensions of our model, including formulating a time-frequency version to handle temporal correlation and replacing the factor model with a mixture of Gaussian distribution to handle non-Gaussianity.

Second, the present algorithm is designed for binary classification tasks. However, in the majority of BCI applications, the user has several separate and distinct, specific intents. For example, in a flight simulator application, in addition to moving the plane left and right, the user may wish to move it up and down, to rotate it at different angles, and to fly it at different speeds. We are therefore extending our model to handle tasks involving more than two classes.

By extracting useful and relevant knowledge from massive information becomes and an essential technique. The MEG BCI paper proposes and utilized the Hadoop Ecosystem based on its massive data management and analysis solutions to catalog better performance on subject’s brainwave data and flight simulator videogame analytics. Additionally, the Hadoop Ecosystem presented data analysis methods based on Pig, HBase and Zookeeper, MapReduce, and HDFS to facilitate as a data warehouse.

For future work, it is also beneficial to investigate mobile applications and cloud-computing integration for multiple uses of the Hadoop Ecosystem as a data store for the MEG BCI tasks and operations by distributing the data across a cluster of nodes (machines).
